# Disparities in Patient Portal Use Among Adults With Chronic Conditions

**DOI:** 10.1001/jamanetworkopen.2024.0680

**Published:** 2024-02-29

**Authors:** Esther Yoon, Scott Hur, Lauren Opsasnick, Wei Huang, Stephanie Batio, Laura M. Curtis, Julia Yoshinso Benavente, Marquita W. Lewis-Thames, David M. Liebovitz, Michael S. Wolf, Marina Serper

**Affiliations:** 1 Center for Applied Health Research on Aging, Feinberg School of Medicine, Northwestern University, Chicago, Illinois; 2 Supportive Oncology, Rush University Cancer Center, Chicago, Illinois; 3 Department of Medical Social Science, Center for Community Health, Feinberg School of Medicine, Northwestern University, Chicago, Illinois; 4 General Internal Medicine, Northwestern University, Chicago, Illinois; 5 Division of Gastroenterology and Hepatology, University of Pennsylvania Perelman School of Medicine, Philadelphia

## Abstract

**Importance:**

Disparities in patient access and use of health care portals have been documented. Limited research has evaluated disparities in portal use during and after the COVID-19 pandemic.

**Objective:**

To assess prevalence of health care portal use before, during, and after the most restrictive phase of the pandemic (2019-2022) among the COVID-19 & Chronic Conditions (C3) cohort and to investigate any disparities in use by sociodemographic factors.

**Design, Setting, and Participants:**

This cohort study uses data from the C3 study, an ongoing, longitudinal, telephone-based survey of participants with multiple chronic conditions. Participants were middle aged and older-adult primary care patients who had an active portal account, recruited from a single academic medical center in Chicago, Illinois, between 2019 and 2022. Data were analyzed between March and June 2022.

**Main Outcomes and Measures:**

Outcomes of portal use (ie, number of days of portal login by year) were recorded for all study participants by the electronic data warehouse. All parent studies had uniform sociodemographic data and measures of social support, self-efficacy, health literacy, and health activation.

**Results:**

Of 536 participants (mean [SD] age, 66.7 [12.0] years; 336 [62.7%] female), 44 (8.2%) were Hispanic or Latinx, 142 (26.5%) were non-Hispanic Black, 322 (60.1%) were non-Hispanic White, and 20 individuals (3.7%) identified as other race, including Asian, Native American or Alaskan Native, and self-reported other race. In multivariable analyses, portal login activity was higher during the 3 years of the COVID-19 pandemic compared with the 2019 baseline. Higher portal login activity was associated with adequate health literacy (incidence rate ratio [IRR], 1.51; 95% CI, 1.18-1.94) and multimorbidity (IRR, 1.38; 95% CI, 1.17-1.64). Lower portal activity was associated with older age (≥70 years: IRR, 0.69; 95% CI, 0.55-0.85) and female sex (IRR, 0.77; 95% CI, 0.66-0.91). Compared with non-Hispanic White patients, lower portal activity was observed among Hispanic or Latinx patients (IRR, 0.66; 95% CI, 0.49-0.89), non-Hispanic Black patients (IRR, 0.68; 95% CI, 0.56-0.83), and patients who identified as other race (IRR, 0.42; 95% CI, 0.28-0.64).

**Conclusions and Relevance:**

This cohort study using data from the C3 study identified changes in portal use over time and highlighted populations that had lower access to health information. The COVID-19 pandemic was associated with an increase in portal use. Sociodemographic disparities by sex and age were reduced, although disparities by health literacy widened. A brief validated health literacy measure may serve as a useful digital literacy screening tool to identify patients who need further support.

## Key Points

**Question:**

Did patient access and use of patient health care portals change during the COVID-19 pandemic, and are there differences by sociodemographic characteristics?

**Findings:**

In this cohort study of 536 participants, significant disparities in portal use by sex, age, multimorbidity, and health literacy were found. While disparities by sex and age decreased and were no longer statistically significant by 2021, disparities by multimorbidity remained consistent throughout the pandemic and disparities by health literacy were exacerbated.

**Meaning:**

These findings suggest that health systems and practices must understand and address persistent disparities in patient portal utilization among some populations (eg, lower health literacy) as they leverage digital health tools.

## Teaser

This cohort study assesses prevalence of and sociodemographic characteristics associated with health care portal use before, during, and after the most restrictive phase of the COVID-19 pandemic (2019-2022).

## Introduction

The COVID-19 pandemic disrupted face-to-face health care delivery and accelerated the adoption and use of digital health modalities, like patient portals.^1,2^ Patient portals are “secure online website that gives patients convenient, 24-hour access to personal health information from anywhere with an internet connection.”^3^ Portal use has been on the rise, given the potential clinical and organizational benefits and activation of provisions from the 21st Century Cures Act, which prohibits information blocking and ensures patients have access to their health data as quickly as possible.^4,5,6^

Prior to the pandemic, there have been known challenges in expanding portal use among members of vulnerable populations (eg, older adults, patients with multimorbidity) who might be considered to benefit the most.^7,8,9,10^ There have also been well-documented disparities: patients with lower socioeconomic status (SES), educational attainment, health literacy, and subsequently those in racial and ethnic minority communities (eg, Black patients) have had lower portal adoption, access, and use.^4,7,11,12^ Studies have also highlighted patient-specific barriers, including concerns around privacy and security, access to technology and internet, limited digital or technology literacy, limited health literacy, and a general preference for the face-to-face modality of care.^9,13^

Less is known about how portal adoption and use have shifted during the COVID-19 pandemic, despite greater health system adoption and meaningful use. Previous studies have evaluated barriers and disparities in portal use in earlier time periods of the pandemic (ie, 2018-2020) or relied on patient self-report.^14,15,16,17^ Although studies have indicated that patients have found portals to be useful tools for managing health during the pandemic, digital literacy was found to be a significant barrier to portal use.^18,19^ Limited information is currently available on the longitudinal trends of portal use during the pandemic era. The few longitudinal studies available have indicated that, although portal use has generally increased with time, sociodemographic disparities (eg, age, sex, race) have persisted.^17,20,21,22^

In this investigation, we evaluated portal use between 2019 and 2022 among a diverse cohort of middle-aged and older adults with at least 1 chronic condition at a large health system. The study aims were to characterize level of portal use, evaluate temporal changes in use, and to examine any sociodemographic disparities in use.

## Methods

This cohort study used data from the COVID-19 & Chronic Conditions (C3) study, approved by Northwestern University institutional review board. As part of their participation in the C3 study, participants provided written informed consent and completed Health Insurance Portability and Accountability Act authorization.

This study was a retrospective cohort study of an ongoing, longitudinal cohort study, the C3 study. The C3 study is a telephone-based survey of participants enrolled in 1 of 5 primary care–based, National Institutes of Health–funded studies (eTable 1 in Supplement 1). The objective was to track the experiences of middle-aged and older adults with underlying health conditions that placed them at higher risk for SARS-CoV-2 infection and adverse outcomes from COVID-19 through the pandemic. C3 parent studies were chosen due to enrollment of participants who would have greater risk for COVID-19 (eg, largely middle-aged or older adult participants, with ≥1 chronic conditions) and included detailed information on sociodemographic characteristics (eg, education, income), health literacy, and patient-reported outcomes that are not routinely collected in clinical care.

To assess the prevalence of C3 participants’ portal use before, during, and after the most restrictive phase of the pandemic and examine sociodemographic disparities in portal use, data from the C3 cohort were matched (using unique patient hospital identification numbers) to data on use and activity of Northwestern Medicine’s patient portal (ie, MyChart; Epic Systems), recorded by the enterprise data warehouse (EDW) between January 1, 2019, and December 31, 2022.

### Measurements

#### Sociodemographic and Psychosocial Characteristics

The C3 study collected self-reported information on patient psychosocial characteristics, COVID-19–related beliefs and actions, health and lifestyle behaviors, health services use, and mental and physical health (eTable 2 in Supplement 1). Depression and anxiety were measured using the respective Patient Reported Outcomes Measurement Information Service short-form instruments, which are validated and normed among the general US population.^23^ For each scale, a raw score was calculated and transformed into corresponding *T*-scores and categorized using the following severity thresholds: none (*T*-score, <55), mild (*T*-score, 55.0-59.9), moderate (*T*-score, 60.0-69.9), and severe (*T*-score, ≥70). For this analysis, we collapsed depression and anxiety measures into 3 severity groups: none (T-score, <55), mild (*T*-score, 55.0-59.9), and moderate or severe (*T*-score, ≥60.0).

All parent studies had uniform collection of patient information via interview, including demographic characteristics (ie, age, sex, self-reported race and ethnicity), SES (ie, household income, number in household, educational attainment, employment status, and health insurance), self-reported chronic conditions, and a 1-item, self-reported overall health question (assessed as excellent, very good, good, fair, or poor). Race and ethnicity were categorized as Hispanic or Latinx, non-Hispanic Black, non-Hispanic White, and other race (eg, Asian, Native American or Alaskan Native, and self-reported other race). In addition, the C3 survey included measures of other factors, including health literacy (measured by the Newest Vital Sign), patient activation (captured with the Consumer Health Activation Index), and tangible social support (assessed with a 2-item validated scale).^24,25,26,27^

#### Portal Use and Activity

Number of days of portal login by year was recorded for all study participants by the EDW. The following portal activities are reported in this study: electronic check-ins, requesting appointments, cancelling appointments, confirming appointments, viewing clinical notes, viewing after-visit summaries, downloading after-visit summaries, checking test results, viewing scans, viewing documents, and patient-clinician messaging. All portal activities were reported by frequency by year (2019-2022).

### Statistical Analysis

Statistical analysis was conducted using RStudio software version 4.3.0 (R Project for Statistical Computing) and Stata/SE software version 18 (StataCorp). Descriptive statistics were conducted on all patient variables. Covariates that were significantly associated in the bivariate analyses were included in the regression model. As the primary outcome, portal login activity, was continuous and nonnormally distributed, we applied a generalized estimating equation with negative binomial regression to model mean change in yearly portal login activity during 2019 through 2022, adjusting for sociodemographic characteristics and year as independent variables. We implemented an autoregressive correlation structure, as we assumed correlations between portal usage are highest between adjacent time points. We used 2019 as our baseline value to compare changes in portal use over time. Incidence rate ratios (IRRs) and estimated probability were reported, with significance set at 2-sided *P* < .05. To determine whether portal activity differed by year across certain sociodemographic characteristics, interaction terms between years and significant variables identified in our initial multivariate model (ie, race, sex, age, multimorbidity [ie, ≥3 chronic conditions], and health literacy) were tested separately. We adjusted these interaction models by race, sex, age, multimorbidity, health literacy, depression, anxiety, and tangible support.

We sought to better understand outliers and conducted an exploratory analysis of frequent portal users, given extreme usage among select participants between 2019 and 2022. We created a new variable of mean portal use between 2020 and 2022 for each participant and reviewed the distribution of mean portal use. We created a dichotomous variable for high portal user (yes or no) using the upper quintile and determined a mean of 69.66 logins to be the minimum threshold to categorize high use (ie, 20% of population that are the highest users). A logistic regression model was fit, adjusting for any covariates associated with high portal use in univariable analysis at *P* < .05, and unadjusted odds ratios (ORs) and adjusted ORs (AORs) are reported. Data were analyzed between March and June 2023.

## Results

Among 718 C3 study participants, 536 (74.7%) had data on portal use (ie, were ever users of the portal) and were included in the study (eTable 3 in Supplement 1). Overall, the mean (SD) age was 66.7 (12.0) years (range, 23 to 91), 336 (62.7%) were female, and 44 (8.2%) were Hispanic or Latinx, 142 (26.5%) were non-Hispanic Black, 322 (60.1%) were non-Hispanic White, and 20 individuals (3.7%) identified as other race (Table 1). Nearly half of the cohort (248 participants [46.3%]) had low patient activation, and 72 participants (13.4%) had limited health literacy. A total of 68 participants (12.7%) had an education level of high school or less, and 59 participants (11.0%) reported living below poverty level. Most participants (339 participants [63.2%]) had multimorbidity.

**Table 1.** Participant Characteristics

Participant characteristics	Patients, No. (%) (N = 536)
Age, y	
Mean (SD)	66.7 (12.01)
<60	123 (22.9)
60-69	161 (30.0)
≥70	252 (47.0)
Sex	
Male	200 (37.3)
Female	336 (62.7)
Race and ethnicity	
Hispanic or Latinx	44 (8.2)
Non-Hispanic Black	142 (26.5)
Non-Hispanic White	322 (60.1)
Other^a^	20 (3.7)
Education level	
≤High school	68 (12.7)
Some college or technical	127 (23.7)
College graduate	341 (63.6)
Employment status	
Not currently working	324 (60.4)
Currently working	180 (33.6)
Below poverty level	
No	471 (87.9)
Yes	59 (11.0)
Health insurance	
Private	145 (27.1)
Medicare or Medicare with private supplement	331 (61.8)
Medicaid or Medicaid with private supplement	59 (11.0)
Limited English proficiency	
No	536 (100.0)
Yes	0
Marital status	
Currently married	220 (41.0)
Not currently married	271 (50.6)
Patient activation	
High	39 (7.3)
Moderate	216 (40.3)
Low	248 (46.3)
Health literacy	
Limited	72 (13.4)
Marginal	111 (20.7)
Adequate	353 (65.9)
Anxiety	
None	369 (68.8)
Mild	94 (17.5)
Moderate or severe	69 (12.9)
Depression	
None	417 (77.8)
Mild	66 (12.3)
Moderate or severe	49 (9.1)
Chronic conditions, No.	
≥3	339 (63.2)
<3	197 (36.8)
Self-reported overall health	
Excellent	71 (13.2)
Very good	191 (35.6)
Good	196 (36.6)
Fair or poor	78 (14.6)
Tangible support	
None needed	442 (82.5)
Adequate	29 (5.4)
Inadequate	61 (11.4)

^a^ Includes Asian, Native American or Alaskan Native, and self-reported other race.

### Portal Use and Activity Over Time

**Figure 1.**
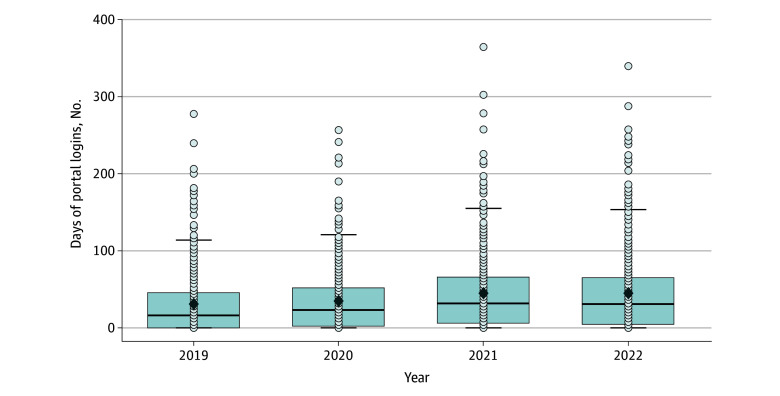
Number of Days of Portal Logins by Each Year All interaction models were adjusted by race, sex, age, multimorbidity, health literacy, depression, anxiety, and tangible support. Dark lines indicate medians, boxes, IQRs; whiskers, 95% CI; dots, individuals data points.

The distributions of portal logins by year are shown in Figure 1. Frequency of portal activity across 2019 to 2022 are reported in Table 2. With respect to the median number of days of portal logins, logins increased from a median (IQR) of 16 (0 to 45.3) days in 2019 to 31 (2 to 52) days in 2020. The median (IQR) number of days of portal logins was 31.5 (6 to 65.3) days in 2021 and 31 (4.8 to 65) days in 2022. The most frequent portal activity was checking laboratory or test results, with a median (IQR) of 4 (0 to 13) logins in 2019 and 2020, 6 (0 to 14) logins in 2021, and 7 (0 to 17) logins in 2022. All other activities, such as scheduling (ie, electronic check-ins or requesting, cancelling, and confirming appointments) and messaging, were low and had medians at or close to 0 across each year.

**Table 2.** Frequency of Portal Activity From 2019 to 2022

Portal activity	Median (range), No.			
	2019	2020	2021	2022
Patient logged in, d	16 (0-277)	31 (0-256)	31.5 (0-364)	31 (0-339)
Electronic check-ins	0 (0-7)	0 (0-25)	0 (0-8)	1 (0-48)
Appointment requests	0 (0-3)	0 (0-3)	0 (0-3)	0 (0-3)
Appointment cancellations	0 (0-11)	0 (0-0)	0 (0-0)	0 (0-0)
Appointment confirmations	0 (0-19)	0 (0-51)	0 (0-22)	0 (0-23)
Clinical note views	0 (0-9)	0 (0-13)	0 (0-37)	1 (0-40)
AVS views	0 (0-2)	0 (0-1)	0 (0-2)	0 (0-1)
AVS downloads	0 (0-1)	0 (0-2)	0 (0-3)	0 (0-1)
Test or laboratory results checked	4 (0-214)	4 (0-109)	6 (0-163)	7 (0-124)
Scan views	0 (0-2)	0 (0-7)	0 (0-1)	0 (0-6)
Document views	0 (0-3)	0 (0-11)	0 (0-36)	0 (0-37)
New conversation messages	0 (0-0)	0 (0-0)	1 (0-47)	4 (0-163)

Abbreviation: AVS, after-visit summary.

### Associations Between Portal Login Activity and Sociodemographic Characteristics

Multivariable results for portal use are reported in Table 3. After adjusting for sociodemographic characteristics, login activity was higher during the 3 years of the COVID-19 pandemic than at the 2019 baseline (2020: IRR, 1.18; 95% CI, 1.12 to 1.25; 2021: IRR, 1.60; 95% CI, 1.48 to 1.72; 2022: IRR, 1.58; 95% CI, 1.45 to 1.73). Higher login activity was associated with adequate health literacy (IRR, 1.51; 95% CI, 1.18 to 1.94) and multimorbidity (IRR, 1.38; 95% CI, 1.17 to 1.64). Participants who were older (≥70 years: IRR, 0.69; 95% CI, 0.55 to 0.85), female (IRR, 0.77; 95% CI, 0.66 to 0.91) had lower portal activity. Compared with non-Hispanic White participants, lower portal use was observed in Hispanic or Latinx participants (IRR, 0.66; 95% CI, 0.49 to 0.89), non-Hispanic Black participants (IRR, 0.68; 95% CI, 0.56 to 0.83), and participants who identified as other race or ethnicity (IRR, 0.42; 95% CI, 0.28 to 0.64). Tangible social support was not associated with login activity.

**Table 3.** Negative Binomial Model Evaluating Association of Sociodemographic Characteristics With Level of Portal Use Over Time

Variables	IRR (95% CI)	*P* value
Year		
2019	1 [Reference]	NA
2020	1.18 (1.12-1.25)	<.001
2021	1.59 (1.48-1.72)	<.001
2022	1.58 (1.44-1.72)	<.001
Age, y		
<60	1 [Reference]	NA
60-69	0.82 (0.65-1.02)	.08
≥70	0.69 (0.56-0.86)	.001
Sex		
Male	1 [Reference]	NA
Female	0.78 (0.66-0.92)	.003
Race and ethnicity		
Hispanic or Latinx	0.66 (0.49-0.89)	.007
Non-Hispanic Black	0.69 (0.57-0.84)	<.001
Non-Hispanic White	1 [Reference]	NA
Other^a^	0.41 (0.27-0.63)	<.001
Health literacy		
Limited	1 [Reference]	NA
Marginal	1.20 (0.92-1.58)	.18
Adequate	1.51 (1.17-1.94)	.001
Anxiety		
None	1 [Reference]	NA
Mild	1.20 (0.98-1.47)	.08
Moderate or severe	1.22 (0.87-1.70)	.25
Depression	1 [Reference]	NA
None		
Mild	0.87 (0.68-1.11)	.25
Moderate or severe	0.77 (0.53-1.13)	.18
Chronic conditions, No.		
≥3	1.38 (1.17-1.64)	<.001
<3	1 [Reference]	NA
Tangible support		
None needed	0.83 (0.65-1.07)	.15
Adequate	0.91 (0.61-1.33)	.62
Inadequate	1 [Reference]	NA

Abbreviations: IRR, incidence rate ratio; NA, not applicable.

^a^ Includes Asian, Native American or Alaskan Native, and self-reported other race.

### Interaction Analyses of Portal Use Over Time

**Figure 2.**
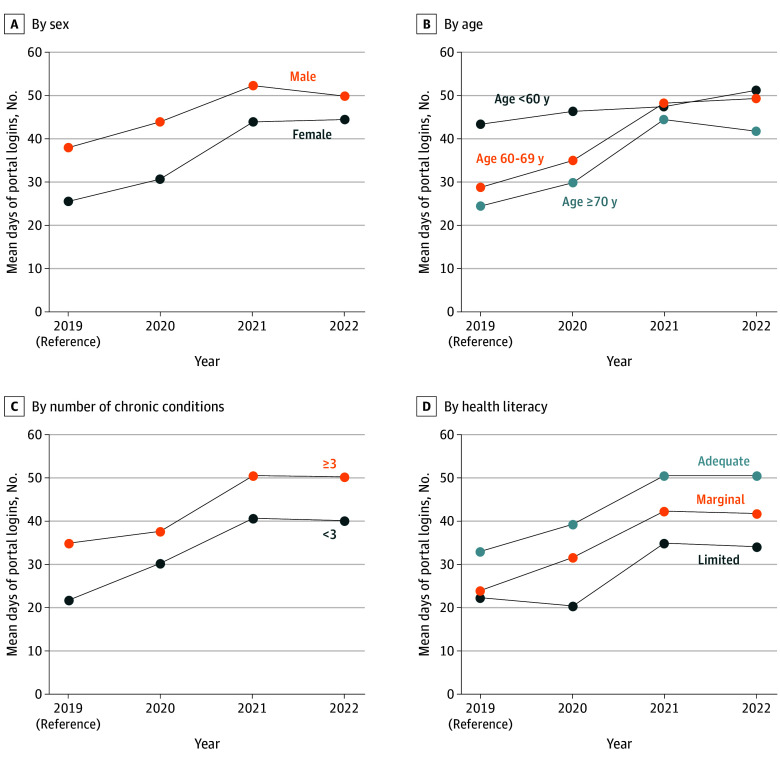
Number of Annual Portal Logins by Sex, Age, Number of Chronic Conditions, and Health Literacy Level

To evaluate for sociodemographic disparities in portal logins over time, interaction terms with year were included separately in the model for race, sex, age, multimorbidity, and health literacy. There was no significant interaction between year and race (χ^2^_9_ = 13.3; *P* = .15); however, significant interactions were noted by sex (χ^2^_3_ = 13.4; *P* = .004), age (χ^2^_6_ = 33.9; *P* < .001), multimorbidity (χ^2^_3_ = 17.5; *P* < .001), and health literacy (χ^2^_6_ = 24.3; *P* < .001). Differences in annual portal usage by these sociodemographic factors are presented in Figure 2.

Women logged in to the portal a mean of 12.35 (95% CI, −18.58 to −6.13) fewer times than men in 2019 (*P* < .001) and 13 fewer times than men in 2020 (*P* < .001). By 2021, this difference by sex was attenuated and no longer significant (adjusted mean difference, −8.1; 95% CI, −17.2 to 0.95; *P* = .08), shrinking further by 2022 (adjusted mean difference, −5.4; 95% CI, −14.2 to 3.5; *P* = .24). Similarly, preexisting differences in portal use by age were reduced. Compared with younger patients (ie, <60 years), older participants (age 60 to 69 and ≥70 years) were significantly less likely to use the portal in 2019 (adjusted mean difference: age 60 to 69 years, −14.57; 95% CI, −24.22 to −4.92; *P* = .003; age ≥70 years, −19.11; 95% CI, −28.32 to −9.90; *P* < .001). By 2021, differences in portal login by age were reduced and no longer significant (adjusted mean difference: age 60 to 69 years, 0.74; 95% CI, −11.38 to 12.84; *P* = .91; age ≥70 years, −3.20; 95% CI, −14.47 to 8.06; *P* = .58).

Patients with multimorbidity logged in to the portal 13.08 (95% CI, 7.92 to 18.24) more times than those without multimorbidity in 2019 (*P* < .001). Disparities in portal use were consistently observed during 2019 through 2022, with the gap narrowing in the peak COVID-19 years of 2020 and 2021. Specifically, compared with patients with fewer chronic conditions, patients with multimorbidity logged into the portal 7.40 (95% CI, 1.13 to 13.68) more times in 2020 (*P* = .02), 9.79 (95% CI, 1.36 to 18.21) more times in 2021 (*P* = .02), and 10.21 (95% CI, 1.87 to 18.55) more times in 2022 (*P* = .02).

Disparities in portal login by health literacy showed a different pattern than what was observed by sex and age. In 2019, patients with adequate health literacy logged in to the portal 10.51(95% CI, 3.72 to 17.31) more times than those with limited health literacy (*P* = .002). In 2020, during the peak of the COVID-19 pandemic, disparities in portal use by health literacy were exacerbated. Compared with participants with inadequate health literacy, patients with marginal health literacy logged into the portal 11.39 (95% CI, 3.58 to 19.20) more times (*P* = .004), and participants with adequate health literacy logged in to the portal 18.94 (95% CI, 12.16 to 25.72) more times (*P* < .001). Furthermore, significant differences in portal login between adequate and limited health literacy persisted into 2021 (adjusted mean difference, 15.77; 95% CI, 5.23 to 26.30; *P* = .003) and 2022 (adjusted mean difference: 16.31; 95% CI, 5.96 to 26.67; *P* = .002).

### Exploratory Analyses of Portal Superusers

In univariable analyses, participants who had high portal utilization were more likely to have adequate health literacy (OR, 2.54; 95% CI, 1.23 to 5.95), mild anxiety (OR, 1.95; 95% CI, 1.15 to 3.24), mild depression (OR, 1.89; 95% CI, 1.04 to 3.34), and multimorbidity (OR, 2.01; 95% CI, 1.26 to 3.28). Participants who were older (ie, ≥70 years: OR, 0.59; 95% CI, 0.35 to 0.98), female (OR, 0.61; 95% CI, 0.40 to 0.93), and non-Hispanic Black (OR, 0.55; 95% CI, 0.32 to 0.92) were less likely to have higher portal use. In multivariable models, mild anxiety (AOR, 2.11; 95% CI, 1.07 to 4.10) and multimorbidity (AOR, 2.29; 95% CI, 1.27 to 4.22) remained independently associated with high portal use (eTable 4 in Supplement 1).

## Discussion

This cohort study highlights longitudinal changes in disparities in patient portal use by key sociodemographic characteristics. Before the COVID-19 pandemic (2019), patients who were female, were older, had fewer comorbidities, or had lower health literacy had significantly fewer portal logins. While disparities associated with sex and age were reduced as the pandemic progressed, disparities by multimorbidity remained and disparities by health literacy were exacerbated, highlighting that populations with pre-existing risk factors, including those with low health literacy, may continue to be left behind in the shift toward digital health.

Our results were consistent with prior literature on digital health inequities among racial and ethnic minority groups, individuals with lower health literacy, and individuals with lower SES, as well as increased portal use among patients with multimorbidity.^28,29,30,31,32,33^ Study results were also similar to more recent health care portal studies that have illustrated increased portal use before and after the most restrictive phase of the pandemic and continued disparities in use (although some studies have reported that disparities attenuated during and after the most restrictive phase of the pandemic).^17,20,21,22,34^ Consistent with other reports, our findings suggest that the pandemic widened disparities in portal use among patients with lower health literacy, who may have more difficulty navigating technology or digital health modalities.^35,36,37^

Our findings also suggest that, compared with younger adults and male patients, older adults and female patients were less likely to use patient portals before the pandemic but had marked increases during the pandemic. While previous studies have found that older adults have lower patient engagement, research has typically found that women have higher levels of patient portal engagement then men.^38,39,40^ Given health disparities in the C3 parent studies, it is possible that C3 participants include a more health care–seeking, proactive male patient population than typical prior studies or a general sample. Another possibility might be caregiver messaging on the patient’s behalf by proxy, which is observed among adults managing chronic conditions.^41,42^ The data for this analysis do not delineate whether portal activity was driven by patients vs caregivers on the patient’s behalf.

Although portal logins increased overall during the pandemic, specific portal activity was limited to reviewing test and laboratory results rather than scheduling, messaging, or viewing other documents, similar to results found in a 2019 study on patient portal use and activity.^43^ Our analyses found that outliers in portal logins (ie, superusers) were more likely to have multimorbidity and mild anxiety.

This study is novel in its analysis of how disparities in the level of portal use evolve over time, providing a unique longitudinal analysis for evaluating sociodemographic differences in portal use during 4 years before, during, and the most restrictive phase of the COVID-19 pandemic among a diverse sample of patients. As telemedicine and digital health continue to evolve, it is important to consider how future directions for health care organizations might address digital health disparities and meaningful use. It may be important to consider how attitudes and perceptions of patient portals might hinder (eg, concerns about privacy and data security) or facilitate portal adoption and meaningful use.^44^

### Limitations

This study had several limitations. Our analysis was reliant on the EDW database and evaluated specific portal activities; therefore, it is unclear whether we fully capture a patient’s portal activity. Our analysis may capture accidental logins or (also known as *phantom logins*) in which a patient logs in and passively views their portal dashboard or notification or message alerts (ie, logging in with no further portal activity completed).

This analysis used a process-based outcome (ie, frequency of annual portal logins) and did not examine whether portal use was associated with better perceived health care quality or improved health outcomes. Logging in to the portal may not equate to meaningful use of the portal or meaningful engagement with health care practitioners in the system.^45^ We were not able to evaluate the association of portal use with clinical decision-making or clinician factors that may be associated with use. Thus, we cannot infer that increased portal logins are a positive outcome. We also did not investigate caregiver or clinician perspectives on portal use. Additionally, analysis did not include portal nonusers, who might be more likely to have barriers in accessing or using healthcare and thereby have poorer health outcomes and face greater health disparities.

Furthermore, generalizability of findings is limited, as the C3 study surveyed patients with underlying health conditions actively enrolled at a single health care system located in 1 large US city. Follow-up investigations are currently underway to examine disparities among C3 participants who sought care in other community health care locations.

## Conclusions

In this cohort study, we include a novel analysis of sociodemographic disparities in portal use over 4 time points before and during the COVID-19 pandemic. Additional research may be warranted to fully understand effective interventions at sitewide and systemwide levels to bridge the gap in portal use and to minimize vulnerable populations continuing to be left behind. Although this study was focused on sociodemographic disparities of portal activity among patients with an active portal account, it is important to consider existing and shifting disparities among individuals who have never used the portal and addressing possible barriers in portal adoption. Furthermore, as telehealth and digital health tools continue to be an integral part of health care systems, future research would benefit from evaluating and optimizing digital literacy challenges as a potential barrier to portal adoption and use, as well as optimizing access to reliable internet or broadband services, particularly for communities that have historically had poor digital access due to limitations in neighborhood infrastructure.

## Notes

Supplement 1. **eTable 1.** C3 Parent Studies **eTable 2.** C3 Study Measures and Outcomes **eTable 3.** Comparison of Patients Without vs Without Patient Portal Accounts **eTable 4.** Bivariate and Multivariable Analyses of Factors Associated With High Portal Utilization

Supplement 2.
Data Sharing Statement
