# Supplementary material for: Disparities in Patient Portal Use Among Adults With Chronic Conditions
Source: JAMA Netw Open. 2024 Feb 29;7(2):e240680. doi: 10.1001/jamanetworkopen.2024.0680 (PMC10905301; doi:10.1001/jamanetworkopen.2024.0680)
Supplement: Supplement 1. — eTable 1. C3 Parent Studies eTable 2. C3 Study Measures and Outcomes eTable 3. Comparison of Patients Without vs Without Patient Portal Accounts eTable 4. Bivariate and Multivariable Analyses of Factors Associated With High Portal Utilization [file jamanetwopen-e240680-s001.pdf]

Supplemental Online Content

Yoon E, Hur S, Opsasnick L, et al. Disparities in patient portal use among adults with chronic conditions. *JAMA Netw Open*. 2024;7(2):e240680. doi:10.1001/jamanetworkopen.2024.0680

eTable 1. C3 Parent Studies
eTable 2. C3 Study Measures and Outcomes
eTable 3. Comparison of Patients Without vs Without Patient Portal Accounts
eTable 4. Bivariate and Multivariable Analyses of Factors Associated With High Portal Utilization

This supplemental material has been provided by the authors to give readers additional information about their work.

© 2024 Yoon E et al. *JAMA Network Open.*

eTable 1. C3 Parent Studies

Parent Study (NIH Project Number)	Design	Eligibility Criteria				Sample		Eligible Pool (N=2,223)
		Age	Language	Clinical	Setting	Chicago (n=673)	New York (n=200)	
Health Literacy and Cognitive Function Among Older Adults (R01AG030611)	Cohort	65–85	English	None	1 academic internal medicine clinic, 5 FQHCs	153	--	776
Self-Management Behaviors among COPD Patients with Multimorbidity (R01HL126508)	Cohort	≥40	English	COPD & htn &/or T2DM	2 academic internal medicine clinics	43	98	168
A Universal Medication Schedule to Promote Adherence to Complex Drug Regimens (R01AG046352)	Clinical Trial	≥50	English & Spanish	Taking ≥5 long-term medications	1 academic internal medicine clinic, 1 FQHC	215	---	459
Transplant Regimen Adherence for Kidney Recipients by Engaging Information Technologies: The TAKE IT Trial (R01DK110172)	Clinical Trial	≥21	English	Kidney transplant	1 organ transplant center	126	---	240
EHR-Based Universal Medication Schedule to Improve Adherence to Complex Regimens (R01NR015444)	Clinical Trial	≥21	English	T2DM, taking ≥5 long-term medications	7 academic internal medicine clinics	136	102	585

COPD = Chronic Obstructive Pulmonary Disease; HTN= Hypertension; T2DM = Type 2 Diabetes Mellitus; FQHC = Federally Qualified Health Center

eTable 2. C3 Study Measures and Outcomes

Variable	Instrument(s) or Measure(s)	Pre-COVID	C3 Cohort
**Sociodemographic and Psychosocial Characteristics**			
Sociodemographic	Age, sex, race/ethnicity, education, employment, household income	•	•
Patient Activation	Consumer Health Activation Index (CHAI)	•	•
Health Literacy	Newest Vital Sign, Brief Health Literacy Screen	•	
Social Support	2-item measure of tangible social support, Martin & Park environmental demands	•	•
Self-Efficacy	Self-Efficacy for Managing Chronic Disease 6 item Scale		•
**COVID-19 Related Beliefs and Actions**			
Awareness & Concern	Perceptions of the virus; feelings about the pandemic		•
Preparedness	Personal preparedness; confidence in state and federal response		•
Actions	Daily routine, change in plans, obtaining prescriptions, leaving the home		•
Testing & Diagnosis	Ability to obtain COVID-19 test, outcome, diagnosis		•
Information-Seeking and Sources	Amount of time per day getting news about COVID-19; source of information		•
**Behavioral Factors**			
Medication Adherence	Ask-12 survey; Proportion of Days Covered (from pharmacy records)	•	•
Harmful Alcohol Use	Alcohol Use Disorders Identification Test (Audit-C)	•	•
Cigarette Smoking	BRFSS Cigarette Smoking	•	•
Nutrition	Rapid Eating Assessment for Participants (REAPS)		•
**Use of Health Information Technology and Health Services**			
Portal Use	Use of portal, type of portal utilization	•	•
Health Information Seeking	HINTS and Pew Research surveys on technology use, information seeking	•	•
Telehealth Experiences	Telehealth visit (Yes/No), satisfaction and preferences for future care		•
Routine & Preventive Services Use	Use of routine and specialty clinic visits, cancer screening, vaccinations	•	•
Urgent Care	ED/Urgent Care Visits or Hospitalizations	•	•
Vaccination	Vaccinated (Yes/No) for flu, shingles, COVID-19 in last 12 months	•	•
**Physical and Mental Health**			
Stress	Cohen 10-item Perceived Stress Scale (PSS-10)		•
Isolation and Loneliness	UCLA 3-item loneliness scale, 1-item loneliness due to COVID-19		•
Sleep Health	PROMIS Sleep Disturbance Short Form 8a		•
Cognitive Complaints	Everyday Cognition (ECog) subscale		•
Anxiety	PROMIS 8-item Anxiety Short-Form	•	•
Depression	PROMIS 4-item Depression Short-Form	•	•
Overall Mental Wellbeing	World Health Organization Five Well-being Index		•
Health Status	PROMIS Physical Function Short-Form, self-reported overall health	•	•
Chronic Disease Outcomes	hemoglobin A1c, blood pressure, cholesterol, EGFR, ACT, and others as relevant	•	•

eTable 3. Comparison of Patients Without vs Without Patient Portal Accounts

**Participant characteristics**	**Patients, No. (%) (N = 718)**		***P* value**
	**Without patient portal account (n = 182)**	**With patient portal account(n = 536)**	
**Age, y**			
**Mean (SD)**	62.73 (7.75)	66.7 (12.01)	<.001
**<60**	67 (36.81)	123 (22.9)	<.001
**60-69**	77 (42.31)	161 (30.0)	
**≥70**	38 (20.88)	252 (47.0)	
**Sex**			
**Male**	51 (28.02)	200 (37.3)	.03
**Female**	131 (71.98)	336 (62.7)	
**Race and ethnicity**			
**Hispanic or Latinx**	76 (41.76)	44 (8.2)	<.001
**Non-Hispanic Black**	65 (35.71)	142 (26.5)	
**Non-Hispanic White**	35 (19.23)	322 (60.1)	
**Other[a]**	6 (3.30)	20 (3.7)	
**Education level**			
**≤High school**	88 (48.35)	68 (12.7)	<.001
**Some college or technical**	45 (24.73)	127 (23.7)	
**College graduate**	49 (26.92)	341 (63.6)	
**Employment status**			
**Not currently working**	110 (60.44)	324 (60.4)	.87
**Currently working**	64 (35.16)	180 (33.6)	
**Below poverty level**			
**No**	74 (40.66)	471 (87.9)	<.001
**Yes**	108 (59.34)	59 (11.0)	
**Health insurance**			
**Private**	12 (6.59)	145 (27.1)	<.001

© 2024 Yoon E et al. *JAMA Network Open.*

**Medicare or Medicare with private supplement**	43 (23.63)	331 (61.8)	
**Medicaid or Medicaid with private supplement**	119 (65.38)	59 (11.0)	
**Limited English proficiency**			
**No**	121 (66.48)	536 (100.0)	<.001
**Yes**	61 (33.52)	0 (0.0)	
**Marital status**			
**Currently married**	47 (25.82)	220 (41.0)	<.001
**Not currently married**	135 (74.18)	271 (50.6)	
**Patient activation**			
**High**	13 (7.14)	39 (7.3)	.42
**Moderate**	69 (37.91)	216 (40.3)	
**Low**	100 (54.95)	248 (46.3)	
**Health literacy**			
**Limited**	80 (43.96)	72 (13.4)	<.001
**Marginal**	52 (28.57)	111 (20.7)	
**Adequate**	50 (27.47)	353 (65.9)	
**Anxiety**			
**None**	0	369 (68.8)	NA
**Mild**	0	94 (17.5)	
**Moderate or severe**	0	69 (12.9)	
**Depression**			
**None**	0	417 (77.8)	NA
**Mild**	0	66 (12.3)	
**Moderate or severe**	0	49 (9.1)	
**Chronic conditions, No.**			
**≥3**	64 (35.16)	339 (63.2)	<.001
**<3**	118 (64.84)	197 (36.8)	

© 2024 Yoon E et al. *JAMA Network Open.*

**Self-reported overall health**			
**Excellent**	6 (3.30)	71 (13.2)	<.001
**Very good**	30 (16.48)	191 (35.6)	
**Good**	71 (39.01)	196 (36.6)	
**Fair or poor**	75 (41.21)	78 (14.6)	
**Tangible support**			
**None needed**	0	442 (82.5)	NA
**Adequate**	0	29 (5.4)	
**Inadequate**	0	61 (11.4)	

Abbreviation: NA, not applicable.

[a]Includes Asian, Native American or Alaskan Native, and self-reported other race.

© 2024 Yoon E et al. *JAMA Network Open.*

eTable 4. Bivariate and Multivariable Analyses of Factors Associated With High Portal Utilization

	Likelihood of high portal utilization		
**Participant Characteristics (n = 536)**	**OR (95% CI)**	**aOR (95% CI)** †	**p**
**Age group**			
< 60 years			
60-69 years	0.76 (0.44, 1.32)	0.87 (0.45, 1.67)	0.68
≥ 70 years	**0.59 (0.35, 0.98) ****	0.94 (0.50, 1.76)	0.84
**Gender**			
Male			
Female	**0.61 (0.40, 0.93) ****	**0.59 (0.36, 0.96) ****	**0.03 ****
**Race**			
Hispanic/Latinx	0.60 (0.24, 1.33)	0.62 (0.23, 1.49)	0.31
Non-Hispanic Black	**0.55 (0.32, 0.92) ****	0.67 (0.36, 1.21)	0.20
Non-Hispanic White			
Other *	0.40 (0.06, 1.44)	0.58 (0.08, 2.41)	0.50
**Education Level**			
HS or less			
Some college or Technical	0.82 (0.38, 1.85)	—	—
College graduate	1.38 (0.73, 2.83)	—	—
**Employment Status**			
Not currently working		—	—
Currently working	1.38 (0.88, 2.15)	—	—
**Below Poverty Level**			
No		—	—
Yes	0.90 (0.43, 1.73)	—	—
**Health Insurance**			
Private		—	—
Medicare or Medicare + Private Supplement	0.68 (0.30, 1.40)	—	—
Medicaid or Medicaid + Private Supplement	1.03 (0.63, 1.65)	—	—
**Marital Status**			
Currently married	1.18 (0.76, 1.83)	—	—
Not currently married			
**CHAI (Patient Activation)**			
High Activation	1.95 (0.89, 4.07)	—	—
Moderate Activation	1.12 (0.71, 1.78)	—	—
Low Activation			
**NVS (health literacy)**			
Limited			
Marginal	1.35 (0.56, 3.52)	1.22 (0.48, 3.27)	0.69
Adequate	**2.54 (1.23, 5.95) ****	2.11 (0.94, 5.29)	0.09
**PROMIS Anxiety**			

© 2024 Yoon E et al. *JAMA Network Open.*

None			
Mild	**1.95 (1.15, 3.24) ****	1.87 (0.99, 3.47)	0.06
Moderate/Severe	1.28 (0.67, 2.35)	1.16 (0.42, 3.09)	0.76
**PROMIS Depression**			
None			
Mild	**1.89 (1.04, 3.34) ****	1.46 (0.68, 3.08)	0.32
Moderate/Severe	1.26 (0.59, 2.50)	1.57 (0.52, 4.73)	0.42
**Number of Chronic Conditions**			
$\geq$ 3	**2.01 (1.26, 3.28) ****	**2.31 (1.35, 4.03) ****	**0.003 ****
< 3			
**Self-Reported Overall Health**			
Excellent			
Very Good	1.34 (0.68, 2.83)	—	—
Good	1.18 (0.59, 2.50)	—	—
Fair/Poor	1.48 (0.66, 3.40)	—	—
**Tangible Support**			
No support needed		—	—
Adequate support	0.81 (0.27, 2.01)	—	—
Inadequate support	1.07 (0.54, 2.01)	—	—

† Multivariable analyses included age, gender, race, health literacy, anxiety, depression, and number of chronic conditions as covariates

* Asian, Native American/Alaskan Native, and self-reported "Other" race

** indicates statistically significant differences between groups (p-value $\leq$ 0.05)

eTable 3. Bivariate and multivariable analyses of factors associated with high portal utilization

© 2024 Yoon E et al. *JAMA Network Open.*
