# Supplementary material for: Disparities in Patient Portal Use Among Adults With Chronic Conditions
Source: JAMA Netw Open. 2024 Feb 29;7(2):e240680. doi: 10.1001/jamanetworkopen.2024.0680 (PMC10905301; doi:10.1001/jamanetworkopen.2024.0680)
Supplement: Supplement 2. — Data Sharing Statement [file jamanetwopen-e240680-s002.pdf]

Data Sharing Statement

Yoon. Disparities in Patient Portal Use Among Adults With Chronic Conditions. *JAMA Netw Open*. Published February 29, 2024. doi:10.1001/jamanetworkopen.2024.0680

Data
Data available: No
